# 

**Published:** 1900-03

**Authors:** 


CONTENTS.

| | PAGE |
|---|---|
| **Notes on Some Thermal, Hydro-Thermal, Electric and Hydro-Electric Procedures, and the Indications for their Use.** By C. J. Whitby, B.A., M.D. Cantab. | 1 |
| **Rheumatic Disease of the Cardiac Muscle.** By Theodore Fisher, M.D. Lond., M.R.C.P. ... ... ... | 16 |
| **Vascular Caruncle of the Urethra.** By A. E. Aust Lawrence, M.D.... | 23 |
| **Non-Bacillary Croup.** By J. O. Symes, M.D., D.P.H. Lond. ... ... ... ... | 25 |
| **The Spontaneous Disappearance of a Sarcomatous Tumour.** By G. Munro Smith, M.R.C.S.... ... ... | 30 |
| **Progress of the Medical Sciences:** | |
| **Medicine.** Theodore Fisher, M.D. | 33 |
| **Surgery.** James Swain ... ... ... | 36 |
| **Ophthalmology.** F. R. Cross ... | 41 |
| **Reviews of Books** ... ... ... ... ... ... | 45 |
| **Notes on Preparations for the Sick** | 72 |
| **Meetings of Societies** ... ... ... ... | 75 |
| **The Library of the Bristol Medico-Chirurgical Society** ... .. ... ... | 85 |
| **Local Medical Notes** ... ... ... ... | 88 |
| **Scraps** ... ... ... ... ... ... ... ... ... | 95 |

